# Miscellaneous

**Published:** 1849-10-01

**Authors:** R. W. S. Lutwidge

**Affiliations:** Chairman; Secretary


### Miscellaneous.

### LETTER TO THE LORD CHANCELLOR FROM THE COMMISSIONERS IN LUNACY,

### WITH REFERENCE TO THEIR DUTIES AND PRACTICE, UNDER THE ACT 8 AND 9 VICT., C. 100.

*Office of Commissioners in Lunacy*, 19, *New-street*, *Spring-gardens*, *July* 4, 1849.

TO THE RIGHT HON. THE LORD HIGH CHANCELLOR.

My Lord—Under ordinary circumstances we should not, in this form, trespass on your lordship's attention. But certain dicta and opinions which have been recently attributed to the Lord Chief Baron of the Exchequer, relative to the law regulating the care and treatment of lunatics, and the duties of the commissioners in that behalf, appear to us so seriously to affect the interpretation and application of the law by which we are governed, that we feel called upon by a sense of duty to point out in this letter to your lordship, (under whose authority more immediately we have the honour to act,) the great evils to society which would ensue from their acceptance and adoption as a practical rule.

The dicta and opinions now referred to are stated to have fallen from the Lord Chief Baron on the trial of a cause, Nottidge v. Ripley, at which his lordship presided, and to have been enunciated, partly by way of comment on the evidence of certain of the witnesses, and partly in his charge to the jury.

They have attracted much public notice, in consequence of the very singular circumstances and history of the case; they are not likely to be subjected to further discussion or review before a higher legal tribunal; and coming as they did from a judge who holds so eminent a position as the Lord Chief Baron, it becomes peculiarly important that we should lose no time in offering such observations thereon as appear to be called for by the circumstances.

In the course of the trial (as reported in the newspapers of the 25th, 26th, and 27th of June last), the Chief Baron stated his opinion to be, that no person ought to be confined in a lunatic establishment, unless "dangerous to himself or others;" and further, that the members of this board were bound to liberate every person not thus dangerous. He also expressed an opinion that a commission of lunacy ought to have been taken out in the case, and intimated that, without such commission, the party confining Miss Nottidge had not "the sanction of the law."

The dicta and opinions here ascribed to the Chief Baron are, we respectfully submit, likely seriously to mislead the medical profession and the public.

We would first refer your lordship to the act of Parliament under which this commission is constituted, and by virtue of which persons of unsound mind are legally placed and detained in licensed houses and other lunatic establishments, for the treatment and cure of their disease.

It will be observed that the object of this act (8 and 9 Vict., c. 100) is the "care and treatment of lunatics" generally, and that it is not limited to any particular class of lunatics, whether dangerous or otherwise. Indeed, the whole tenour of this and of the County Asylums Act (8 and

9 Vict., c. 126) shows that these acts extend to lunatics of every description, and that dangerous lunatics are only occasionally noticed, where it is necessary to except and distinguish them from the rest. The act, as set forth in its title, is, "An Act for the Regulation of the Care and Treatment of Lunatics;" and the word "lunatic" is (s. 114) defined to mean "every insane person and every person being an idiot or lunatic, or of unsound mind;" and in the statement annexed to the order authorizing the patient's confinement, one point of inquiry is in these words:—"Whether suicidal or dangerous to others;" thereby denoting that patients who are not included in that class are equally subjected to the provisions of the act. The same observation applies to the County Asylums Act (8 and 9 Vict., c. 126), where (ss. 27 and 47, and schedule D) dangerous lunatics are also referred to as forming part only of the body of insane persons, whose confinement and treatment in lunatic asylums are thereby authorised.

The object of these acts is not, as your lordship is aware, so much to confine lunatics, as to restore to a healthy state of mind such of them as are curable, and to afford comfort and protection to the rest. Amongst the many persons confined as being lunatics or of unsound mind, those who are manifestly dangerous, that is to say, those who, by some overt act, have already proved themselves to be dangerous, are comparatively few in number; the far more numerous classes consisting of—1. Those who are sent into lunatic establishments for the purpose of treatment, with a view to the alleviation and cure of their malady; 2. Those who, from disease of mind, are incapable of self-government, and who therefore require, at certain periods, (or perhaps generally,) the most careful supervision and control; and 3. Those who are incapable of taking care of themselves or their affairs, and are likely, therefore, to sustain serious injury if left at large and unprotected.

It may reasonably be asked, what would become of all these large classes of the insane if set at large, in conformity with the Lord Chief Baron's opinion?

It is well known that all commissions of lunacy are founded, not on the party being dangerous, but on the fact of his being insane, and incapable of managing his own affairs; and yet, every one who is found lunatic by inquisition is placed under the care of a committee of his person, who thereupon possesses the entire control over the lunatic, and may keep him in confinement so long as the commission remains in force.

If, in practice, the class of insane persons placed in confinement were limited to such as had previously exhibited some dangerous tendency, the main purposes of the legislature, in the statutes now in force, would be frustrated, and a most fearful hazard be incurred. For, inasmuch as the tendency to danger first shows itself more frequently in the latter than in the earlier stages of the disease, when alone such disease is likely to be cured, a large proportion of patients of this class would be deprived of the benefit of proper curative treatment until after they had placed either themselves or other persons in peril, and had not improbably (owing to the lapse of time) become themselves incurable.

Moreover, the difficulty of ascertaining whether one who is insane be dangerous or not is exceedingly great, and, in some cases, can only be determined after minute observation for a considerable time. It is the general opinion of experienced persons, that whenever an insane delusion exists, the patient can in no case be considered as otherwise than dangerous, although the tendency may never have been actually exhibited by overt acts or expressions; and, in our own experience, we have known patients whose disorder has appeared to have abated, and who have been treated as harmless for a considerable time, but who, nevertheless, upon some sudden and apparently unprovoked impulse, and without betraying any preliminary violence or irritation, have attempted, and in some instances have effected, the destruction of themselves or others.

In the cases of monomaniacs, and patients suffering under religious and other delusions, (not apparently tending to any dangerous result,) we have known repeated instances of their attempting and committing self-destruction, homicide, and acts of violence, owing to some imaginary sentence of condemnation, or under the influence of some imaginary voice or spirit.

In the majority of cases which come under the cognizance of the commissioners, they have little difficulty in satisfying themselves as to the state of mind of the patient; but cases of nicety and difficulty occasionally arise, exhibiting such peculiarities, and differing so decidedly in some respects from all others, that the commissioners, in dealing with them, have been unable to lay down any general rule or principle for their guidance. In no case have they decided that opinions, however wild or extravagant, which were common to any class or body of persons, either in reference to religious belief or otherwise, constituted or amounted to insanity. And in no case have they decided that a patient was insane because his symptoms resembled, in a greater or less degree, those of other patients whom they have previously known; but they have considered each individual case as depending upon its own special circumstances, and have formed their judgment accordingly.

We now beg to address a few words to your lordship with respect to the powers and duties of the members of this commission, a matter which appears to have been much misunderstood.

Amongst the multifarious duties of the commissioners, one is, to discharge patients when "detained without sufficient cause;" of the sufficiency of the cause the commissioners are by law constituted the judges; and they endeavour to form the best judgment in their power in each particular case, when the subject comes before them. Thus, if a patient be placed in an asylum without just cause, without having been of unsound mind, the commissioners are bound to discharge him. So, if a patient (originally a fit subject for confinement) be recovered and restored to a sound state of mind, the commissioners are obviously bound to discharge him, if the person under whose care the patient is placed, or the relative who originally authorized his confinement, (for upon these primarily the duty devolves,) has neglected to do so. But a person labouring under an insane delusion is not detained without sufficient cause.

Under peculiar circumstances, where, after sufficient observation, a patient, although of weak or unsound mind, appears to be perfectly harmless, the commissioners frequently promote his liberation, if he have a comfortable home, or any friends disposed to receive and protect him and his property from injury; but, where this is wanting, the commissioners do not think themselves justified in removing the patient from the shelter of an asylum, and leaving him at large and unprotected. They consider it to be quite clear that they are not bound, as a general rule, to speculate upon the chance that a patient, who, in their opinion, is still insane, will be perfectly harmless if at large, and therefore to liberate him accordingly.

The person signing the order for a patient's confinement (generally a relative or friend) not unfrequently, indeed, takes upon himself the responsibility of liberating a patient whilst still under a delusion, and before recovery, and the commissioners have no right, and never attempt to interfere. The consequence of the premature discharge of a lunatic patient, however, is frequently a relapse, and should, as much as possible, be avoided. Even with all the caution now exercised by the medical officers of asylums, many of whom possess great experience, and have daily opportunities of watching the process of recovery, it has been found necessary to re-admit, within a short period, many patients whom they have discharged as recovered.

The power of liberation vested in the commissioners is one involving great responsibility, and, in our judgment, ought to be exercised only after grave consideration, and with much caution. Every person placed in confinement as a lunatic must *primâ facie* be presumed to be insane. Before a private patient can be legally detained in any house, there must exist an order (signed by some friend or relative), two certificates from different medical practitioners, who have each separately examined the patient, and also a third certificate or statement from the medical officer of the establishment, all expressing the condition of the individual as of unsound mind, and a proper subject for confinement. It would argue great rashness and imprudence, to say the least, on our parts, to determine on the immediate, or even the very speedy liberation of a person so certified, unless we had reason to suppose that the certificates had been fraudulently obtained, or we were strengthened in our own impressions by the opinion of the medical officers having the care of the patient, that the confinement had from the first been improper, or that the nature of the malady was such as is usually of short duration, and that a perfect cure had been effected. Although, in a few cases of acute mania, the disorder is sometimes of short duration, yet where there exist actual delusions, the process of recovery (if ever it takes place) is slow and gradual, and the question as to the probability of cure can scarcely be determined satisfactorily until after a considerable period has elapsed.

The opinion attributed to the Lord Chief Baron, that a commission is necessary in all cases, in order to give the confinement the sanction of the law, appears to call for some remark. It is hardly necessary to observe, that proceedings by commission are, generally speaking, advisable only where the insanity is likely to be of a permanent character, and the property of the lunatic is of such a nature as to require them, and of an amount adequate to meet the expense, always considerable, and, when the commission is contested, frequently very large.

Wherever a reasonable hope of recovery exists, and the income of the lunatic can, in the meantime, be properly administered for his benefit without a commission, the general practice, amongst the friends and relatives of the insane is to avoid resorting to proceedings which entail unnecessary cost—which, by the disclosures they occasion, are most painful to the feelings of the family—and which, by the excitement they produce, are sometimes injurious to the patient himself.

It is obvious that the finding of a jury is in no case essential, in order legally to justify the confinement of a person of unsound mind. In fact, out of 4028 private patients (many of them possessed of considerable property), who were confined in asylums on the 1st of January, 1848, only 245 had been found lunatic by inquisition.

To revert to the opinion stated to have been expressed by the Chief Baron, that no person should be placed or detained in any lunatic asylum, unless he be dangerous to himself or others,—upon this point it is of vital importance that no mistake or misconception should exist, and that every medical man, who may be applied to for advice on the subject of lunacy, and every relative and friend of any lunatic, as well as every magistrate and parish officer, (each of whom may be called upon to act in cases of this sort,) should know and be well assured, that according to law, any person of unsound mind, whether he be pronounced dangerous or not, may legally and properly be placed in a county asylum, lunatic hospital, or licensed house, on the authority of the preliminary order and certificates prescribed by the Acts 8 & 9 Vict. c. 100, or c. 126 (as the case may be.)

The order and certificates thus obtained show that the person mentioned therein is either a lunatic, an idiot, or a person of unsound mind, and a proper person to be confined, and fully justify all parties in the matter; and they enable the proprietor or superintendent of any hospital or licensed house to plead them in defence to any action, and are, in the words of the statute, a justification for "taking, confining, detaining, or retaking" the patient, (see 8 & 9 Vict. c. 100, s. 99.)

If all lunatics and persons of unsound mind, except such as had previously manifested a dangerous tendency, were to be excluded from the care and treatment provided in lunatic establishments, sanctioned by law, for the benefit of the whole class, the most lamentable consequences must ensue.

In respect to pauper lunatics, it has already been the subject of almost universal complaint, that the number of such lunatics has been multiplied, and the country burdened to a prodigious amount, because the poorer class of lunatics have been allowed to remain at large, or kept in workhouses, deprived of that medical treatment which a lunatic establishment, properly managed, is best calculated to afford, until their malady has become incurable.

The misery to lunatics' families, and the great cost to the various parishes and counties, consequent on this course, it would be difficult to exaggerate.

In regard to private patients, if not placed for cure or care in some lunatic establishment, they must be kept at home under every disadvantage to themselves, and be the cause of great and unnecessary expense, and of inexpressible annoyance to their families. The first, and an essential proceeding, with a view to cure, is, generally, to detach the patient from the scenes and associations in the midst of which his disorder has arisen. If he were to remain at home, this could not be effected; the proper treatment and accommodation could not be obtained, inasmuch as separate apartments, separate attendants, and daily medical supervision, are necessary—and these, in ninety-nine cases out of a hundred, would be beyond the means of the patient's family to afford. Again, the habits and general conduct of patients under the influence of mental disease are frequently so violent, and at times so offensive, that it would be to the last degree cruel and unjust to expose the other members of the family to them; more especially where there are children, whose minds might receive a shock, and perhaps be incurably injured, by continually witnessing the paroxysms or maniacal extravagances of a lunatic. Equally unjust would it be to suffer the infirmities of the patient himself to be exposed to the gaze of all the members of the household, and, in many cases, to the notice and comments of the neighbourhood and of strangers. There are cases of insanity, as your lordship is aware, in which the most distressing symptoms appear, —in which the character of the individual for a time becomes altogether distorted—his habits filthy—his expressions and general conduct disgusting. There are also cases of females suffering under a form of mental disorder well known to the medical profession, in which, from disease, not only the words, but the actions, also, of the patient become absolutely uncontrollable, where the original and real character is, for a time, altogether subverted, and all modest restraint and decency are abandoned. The want of moral control, indeed, is one of the most common symptoms and indications of insanity; and the actions and expressions of a large number of patients, suffering, at certain periods, under maniacal excitement, are of such a nature that it becomes an imperative duty to protect and shield them from observation as much as possible. The privacy indispensable in cases of this sort can only be properly afforded in houses adapted for the purpose of receiving lunatics, who there, at least, are secluded from the observation of all persons except those under whose care they are immediately placed, and are generally exempted from that mechanical restraint or coercion of the person, which, if they were confined at their own homes, must frequently be inevitable.

There are cases, also, where the presence and example of the patient, if at home, would probably lead to the most distressing and dangerous consequences amongst other members of the family—more especially amongst those of a sensitive and nervous temperament. These remarks respecting private patients apply (as, indeed, is obvious) not merely to those who are dangerous to themselves or others, but to all insane persons whatsoever, of every class and character, including many who, so far as their acts are concerned, might be denominated harmless.

Persons of unsound mind, therefore, whether dangerous or not, are placed in lunatic establishments; some remain there after they are apparently much relieved, because their disease is of a recurrent nature. Others remain there, who, although labouring under insane delusions, are apparently harmless, and generally well conducted, so long as they are under proper control and supervision, but who exhibit their former insane propensities and infirmities the moment that this control and supervision are withdrawn. There are numerous cases, within our recollection, where patients thus circumstanced have been taken out of lunatic establishments upon trial, but who, after a short intercourse with the world, have again exhibited maniacal excitement, or mental incapacity, and have, for the sake of their own safety and welfare, been re-admitted.

We have felt it our duty to submit the foregoing observations to your lordship, for the purpose of putting you in possession of our views and opinions, as set forth in this letter, and of explaining to your lordship the course which we have hitherto pursued, and which we feel bound to continue to pursue, in the exercise of the delicate and responsible functions entrusted to us by the Legislature.

On behalf of the Commissioners in Lunacy.

Ashley, Chairman.

R. W. S. Lutwidge, Secretary.

---

